# Management of a neurotrophic deep corneal ulcer with amniotic membrane transplantation in a patient with functional monocular vision: A case report

**DOI:** 10.1097/MD.0000000000008997

**Published:** 2017-12-15

**Authors:** Tobias Röck, Karl Ulrich Bartz-Schmidt, Daniel Röck

**Affiliations:** Centre for Ophthalmology, University of Tübingen, Tübingen, Germany.

**Keywords:** amniotic membrane transplantation, cornea, corneal ulcer, ocular surface reconstruction, regeneration

## Abstract

**Rationale:**

Amniotic membrane transplantation (AMT) has been performed therapeutically in humans for over 100 years. In recent 2 decades AMTs have been used increasingly and successfully to treat various types of ophthalmic indications.

**Patient concerns:**

An 83-year-old man was referred to our eye hospital with a refractory neurotrophic deep corneal ulcer of the left eye.

**Diagnoses:**

The best-corrected visual acuity of the left eye was 0.5 (0.3 logMAR) and of the right eye was 0.05 (1.3 logMAR), which was caused by a central retinal vein occlusion 5 years previously. In cases of binocular vision, a large amniotic membrane patch can cover the whole cornea, including the optical axis. However, in cases with functional monocular vision, as in the case reported here, the AMT has to be performed without the involvement of the optical axis to ensure vision for the patient. Otherwise the patient would have a massively restricted view like looking through waxed paper for at least 2–4 weeks until the overlay dissolved.

**Interventions:**

For this case, an AMT using a modified sandwich technique was applied without involvement of the optic axis to ensure vision for the patient. This case report illustrates this eye's course of healing over time.

**Outcomes:**

A reduction in the inflammation and healing of the corneal ulcer could be seen. In addition, the corneal vascularization decreased. Six months after the AMT, a slit-lamp examination revealed stable findings. The best-corrected visual acuity of the left eye had increased to 0.8 (0.1 logMAR).

**Lessons:**

To the best of our knowledge, a case report on the management of a neurotrophic deep corneal ulcer with AMT in a patient with functional monocular vision has never been undertaken before.

## 1 Introduction

Amniotic membrane transplantation (AMT) has been performed therapeutically in humans for over 100 years as a skin substitute for treating open wounds.^[1]^ In 1940, de Rotth^[2]^ described the first clinical use of an amniotic membrane (AM) in ophthalmology. He used a fresh AM as a biological bandage material for the management of conjunctival defects.

The AM, which is the inner layer of the placenta, is a thin, semitransparent, resilient, and avascular tissue, which consists histologically of a single epithelial layer, a thick basement membrane, and an avascular stroma. The AM contains abundant growth factors, mitogenic factors, antiangiogenic factors, anti-inflammatory proteins, natural protease inhibitors, and antiscarring properties.^[3]^ In recent years, many studies have shown the clinical efficacy of AMT in stimulating wound healing by promoting epithelialization while suppressing inflammation, angiogenesis, and scarring.^[3,4]^ The AM not only facilitates healing, but also supports regeneration.^[5]^

AMTs have been used increasingly and successfully to treat various types of ophthalmic indications, which include chemical or thermal burns, persistent corneal epithelial defects, corneal ulcers, the reconstruction of conjunctival and ocular surfaces, ocular pemphigoid or Stevens–Johnson syndrome, and bullous keratopathy.^[6–10]^ Here, we report the case of a neurotrophic deep corneal ulcer in a patient with functional monocular vision managed using an AMT.

## 2 Case report

An 83-year-old man was referred to our eye hospital in February 2017 with a refractory neurotrophic deep corneal ulcer of the left eye. This was caused by lagophthalmos as a result of facial paralysis after undergoing surgery for an acoustic neuroma 25 years previously. This corneal ulcer had been previously treated with artificial tears, prophylactic antibiotic eye drops (0.5% moxifloxacin hydrochloride ophthalmic solution), a bandage contact lens, and a watch glass bandage without success.

**Figure 1:**
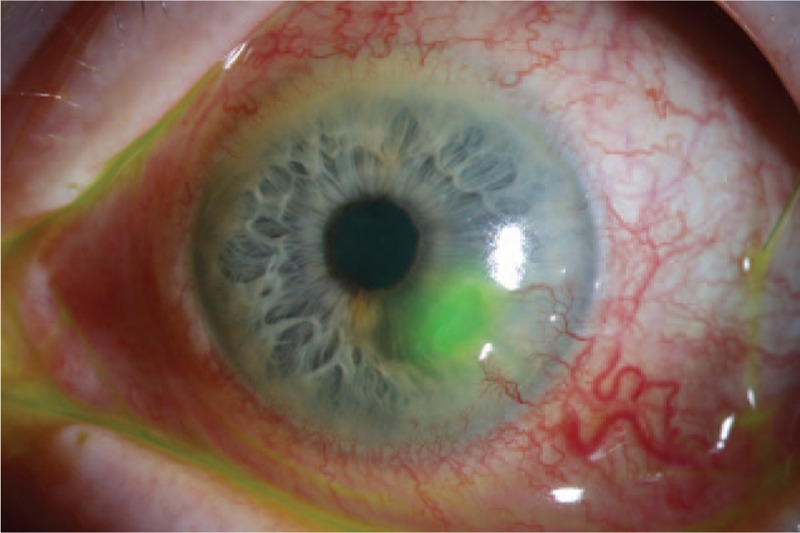
Initial slit-lamp image showing a fluorescein-stained deep paracentral corneal ulcer with corneal vascularization and ocular surface inflammation.

The best-corrected visual acuity of the left eye was 0.5 (0.3 logMAR) and of the right eye was 0.05 (1.3 logMAR), which was caused by a central retinal vein occlusion 5 years previously. The initial clinical findings showed a persistent deep corneal ulcer in the left eye with functional monocular vision (Fig. 1).

**Figure 2:**
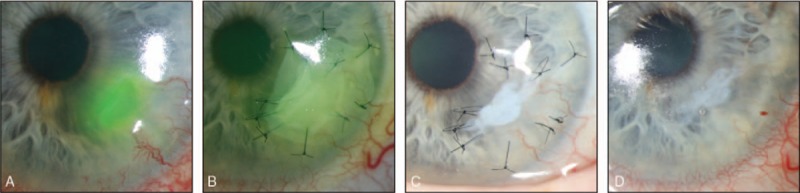
Slit-lamp images showing the course of healing over time of the corneal ulcer. (A) Fluorescein-stained deep corneal ulcer before surgery. (B) Three days after AMT without involvement of the optic axis to ensure vision for the patient. (C) Three weeks after the AMT. (D) Five weeks after the AMT, after removing the corneal sutures. AMT = amniotic membrane transplantation.

For this case, an AMT using the sandwich technique was applied, and Figure 2 shows this eye's course of healing over time. After the AMT procedure, the corneal ulcer was treated with artificial tears, a bandage contact lens, and antibiotic eye drops (0.5% moxifloxacin hydrochloride ophthalmic solution) 4 times a day for 2 weeks. A reduction in the inflammation and healing of the corneal ulcer could be seen. The corneal epithelium grew under the patch but over the uppermost part of the graft, so the AM became partly integrated into the host tissue. In addition, the corneal vascularization decreased.

**Figure 3:**
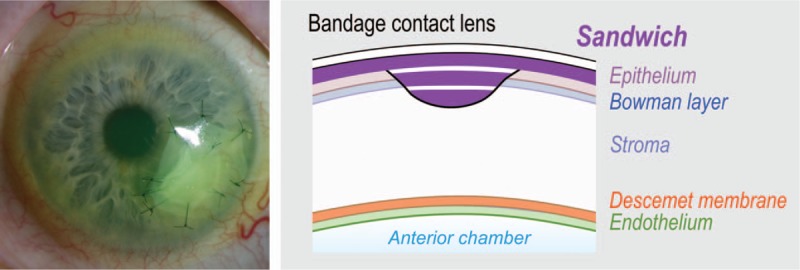
Slit-lamp image with a schematic overview displaying the sandwich technique of AMT. It is a combination of the inlay (graft) and overlay (patch) techniques. The sandwich technique is used for deep stromal defects with large nonhealing epithelial defects. The corneal epithelium is expected to grow under the patch but over the uppermost part of the graft. AMT = amniotic membrane transplantation.

The sandwich technique is used for deep stromal defects with large nonhealing corneal defects and is a combination of the inlay (graft) and overlay (patch) techniques (Fig. 3).

The inlay or graft technique is used for stromal defects, in which the graft is fixed on the stromal defect with single button 10.0 nylon sutures at the periphery of the corneal ulcer. Before transplantation, a small rim of deepithelialization around the stromal defect is prepared to ensure that no epithelium remains under the graft. The epithelium is expected to grow over the AM, which provides a new basement membrane. The overlay or patch technique is used for corneal diseases with nonhealing epithelial defects having no or only shallow stromal defects, for example, after a chemical or thermal burn or recurrent corneal erosions. In this technique, the AM is sutured over the peripheral epithelial remnants and the centrally denuded stroma.

**Figure 4:**
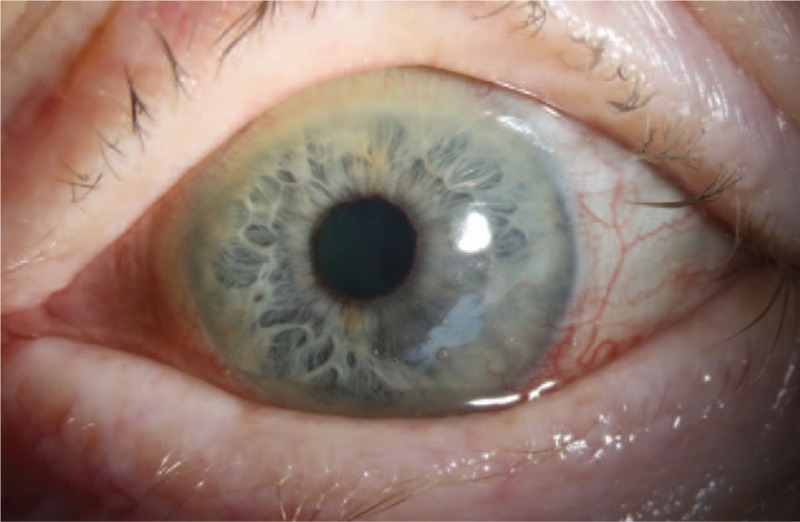
Six months after the AMT, a slit-lamp examination revealing stable findings when compared with the previous visit. The AM has been partly integrated into the host tissue, while the ocular surface inflammation and corneal vascularization have decreased. AM = amniotic membrane, AMT = amniotic membrane transplantation.

Six months after the AMT, a slit-lamp examination revealed stable findings when compared with the previous visit (Fig. 4). The best-corrected visual acuity of the left eye had increased to 0.8 (0.1 logMAR). The consent of the patient was obtained to publish this case report. This study was approved by the Institutional Review Board of the University of Tübingen and adhered to the tenets of the Declaration of Helsinki.

## 3 Discussion

A neurotrophic corneal ulcer is a degenerative disease caused by an impairment in the trigeminal innervation leading to corneal sensitivity reduction, spontaneous epithelial breakdown, and corneal healing impairment.

An AMT has been found to be a good method for corneal reconstruction and regeneration in many clinical situations, including burns, persistent epithelial defects of the cornea, corneal ulcers, and diseases of the conjunctiva, when conservative treatment methods fail.^[11]^ An AMT can even be used to reconstruct and restore the conjunctival surfaces after the resection of a large ocular surface neoplasm.^[12]^ It can also help to maintain a normal conjunctival phenotype and possesses cosmetic benefits over buccal or mucosal grafts, which result in a nonconjunctival epithelial morphology.^[13–15]^

The AM provides a rich source of stem cells along with its unique features; it supports, facilitates, and promotes conjunctival and corneal epithelialization and healing, and inhibits and allows reductions in inflammation, immune rejection, vascularization, scarring, and pain.^[3–5]^ AM-induced pain relief has been clinically shown in the management of chemical burns,^[16,17]^ severe bacterial keratitis,^[18]^ Steven–Johnson syndrome,^[19,20]^ and painful bullous keratopathy.^[21–24]^ The great widespread use of AMTs is likely influenced by the easy processing and cryopreservation techniques, which were introduced 20 years ago.^[25]^

AMT has brought about major advances in ocular surface reconstructive surgery. It can be used as a permanent graft for a tissue defect, so that the epithelial cells grow over the AM, and the membrane will subsequently be integrated into the host tissue (e.g., the corneal stroma), which helps to improve the structural quality of the tissue. The main target of this technique is to provide stability for the regenerating epithelium and restore the tissue integrity and function. In this mode, the inlay or graft can be used as a single layer or multilayers and fixed with single-button 10.0 nylon sutures at the periphery of the corneal ulcer. When the AM is used as a temporary biological patch or overlay, the main goals are to suppress inflammation, reduce scarring, decrease vascularization, and promote healing. The AM is sutured to the ocular surface using a patch that is larger than the defect. In this way, the AM acts as a biological bandage.

As shown in this case, sometimes both an inlay and an overlay are used together in a sandwich technique, in which case the overlay is used as a protective shield to ensure the epithelialization of the AM that is used as the inlay.^[26–28]^ In this case, the epithelium was expected to grow between the uppermost inlay and the patch.

In cases of binocular vision, a large AM patch can cover the whole cornea, including the optical axis. However, in cases with functional monocular vision, as in the case reported here, the AMT has to be performed without the involvement of the optical axis to ensure vision for the patient. Otherwise the patient would have a massively restricted view for 2–4 weeks until the overlay dissolved. These points must always be discussed with the patient before proceeding with an AMT.

## 4 Conclusion

Those patients suffering from persistent corneal defects may benefit from an AMT. Here, our case report showed the healing of a persistent deep corneal ulcer after an AMT using the sandwich technique. In cases with functional monocular vision, an AMT must be performed with modified surgical techniques to ensure vision for the patient. The increasing popularity of AMTs in recent years has likely been influenced by the introduction of different surgical techniques, the rising knowledge about growth factors, neurotrophins, and cytokines, and demographic changes.
